# Physical Therapists’ Self-Reported Perceptions of Bedside Manners in Saudi Arabia: A Mixed-Methods Study

**DOI:** 10.3390/healthcare14152250

**Published:** 2026-07-23

**Authors:** Ali Alsouhibani, Faris M. Aba-Alkhayl, Moodhi M. Alfouzan, Sara H. Althinayyan, Ryhana M. Alnoshan, Jana B. Albarrak, Wasan A. Alsaheel, Fayzah A. Almohaimeed, Alanoud I. Alsughayyir, Raghad Aljutaily, Ruba S. Alquraishi, Renad K. Aldughaim, Fai Alqazlan, Saleh M. Aloraini

**Affiliations:** 1 Department of Physical Therapy, College of Applied Medical Sciences, Qassim University, Buraydah 51452, Saudi Arabia; modyalfowzan@gmail.com (M.M.A.); pt.sarathinayyan@gmail.com (S.H.A.); ryhana.alnoshan@gmail.com (R.M.A.); janaalbarrak@gmail.com (J.B.A.); walsaheel@moh.gov.sa (W.A.A.); fayzahazizz@gmail.com (F.A.A.); nmalanoud@gmail.com (A.I.A.); ra.aljutaily@gmail.com (R.A.); rubaalquraishi22@gmail.com (R.S.A.); renadaldughaim@gmail.com (R.K.A.); fay3li.fa@gmail.com (F.A.); saloraini@qu.edu.sa (S.M.A.); 2 Department of Occupational Therapy, College of Medical Rehabilitation Sciences, Taibah University, Madinah 42353, Saudi Arabia; fabaalkhayl@taibahu.edu.sa; 3 Rehabilitation Programs and Services Department, Sultan Bin Abdulaziz Humanitarian City, Riyadh 11536, Saudi Arabia; 4 Kheraif Physical Therapy Clinic, Buraydah 52387, Saudi Arabia; 5 Qassim Health Cluster, Buraydah 52366, Saudi Arabia; 6 Rehabilitation Department, Ministry of the National Guard-Health Affairs, Riyadh 11426, Saudi Arabia

**Keywords:** bedside manners, physical therapy, patient care, mixed-methods, Saudi Arabia, Ethics

## Abstract

**Highlights:**

**What are the main findings?**

- Saudi physical therapists showed strong support for bedside manners, especially introducing themselves, listening carefully, obtaining consent, maintaining privacy, and reassuring patients during care.
- Qualitative interviews highlighted trust-building, body language, balanced empathy, therapist presence, and equitable patient care as key elements of bedside manners.

**What are the implications of the main findings?**

- Bedside manners should be reinforced in physical therapy education and continuing professional development to support more consistent patient-centered care.
- Embedding bedside manners in professional standards and self-assessment may help physical therapists apply communication, empathy, consent, and privacy consistently in their own practice.

**Abstract:**

Background/Objectives: Bedside manners are an important part of patient-centered physical therapy care, yet little is known about how Saudi physical therapists understand and apply them in practice. This study examined therapists’ perceptions of bedside manners and ethics, and explored differences related to gender and work setting. Methods: A cross-sectional mixed-methods study was conducted among 147 licensed Saudi physical therapists. Quantitative data were collected using a 28-item questionnaire, and qualitative data were gathered through semi-structured interviews with 22 participants. Survey responses were summarized using frequencies and percentages, and differences by gender and work setting were reported. Interview data were analyzed using Braun and Clarke’s thematic analysis. Results: Most participants were female (66.7%) and aged 20–30 years (70.7%). Overall, therapists reported very high agreement with bedside-manner principles across most items. Descriptive patterns by gender and work setting in some perceptions (e.g., patient privacy, therapist presence, and attention to minor cases) were noted. Qualitative analysis identified seven themes: building trust, perceived positive contribution to care, body language, differing views on empathy, therapist presence, patient equity, and seeking help from colleagues. Conclusions: Saudi physical therapists strongly supported core bedside manner behaviors, especially communication, privacy, reassurance, and fair treatment of patients. These findings may inform efforts to incorporate bedside manners into physical therapy education and professional development in support of ethical, patient-centered practice.

## 1. Introduction

Bedside manners represent an essential concept that captures doctors’ approach to patient engagement, emphasizing their manner and style of interaction [1]. It refers to the ways healthcare practitioners communicate with patients—verbally and non-verbally— while demonstrating empathy and maintaining professional conduct [2]. Effective patient–practitioner interaction is fundamental during treatment process [3], as strong communication and interpersonal skills enable practitioners to collect accurate patient histories, conduct appropriate examinations, and implement effective treatment plans [4]. Positive bedside manners strengthen the patient–practitioner relationship and encourage adherence to educational strategies and prescribed treatments [5,6]. Evidence shows that collaborative communication grounded in shared decision-making [7], patient-centeredness [8], and clear information [9] leads to improved patient satisfaction, better disease understanding, enhanced adherence, and improved health outcomes [10,11,12,13].

In physical therapy settings, positive patient–therapist interaction is associated with reduced pain, increased treatment satisfaction, and reduced disability [14,15,16]. Several factors support effective communication and improved treatment outcomes, including listening, empathy, friendliness, encouragement, confidence, and nonverbal communication [17]. Empathy is a core aspect of the patient–therapist relationship [17,18,19,20], and its absence can create a significant barrier to effective interaction [21]. Motivation and encouragement demonstrate therapists’ investment in patient care and reinforce the therapeutic relationship [22,23], while also promoting adherence to treatment and continued improvement [17,23,24]. Both patients and therapists recognize non-verbal communication as a vital component of effective interactions, as attentive therapist behavior increases patients’ comfort and sense of support [25,26] and provides insight into their needs, emotions, and responses [19,27].

Lack of communication between therapists and patients contributes to negative treatment outcomes [28], and ineffective listening, along with the absence of a personal manner, plays a significant role in rehabilitation failure [29]. Patients often feel upset when therapists interrupt them or prevent them from telling their story [21,25,30]. Being seen, heard, and believed is essential to the quality of interaction [31], while rushed behavior or disregarding patients’ opinions weakens the patient–therapist relationship [23,24,25,32]. Conversely, patients value when therapists take time to clearly explain their condition and treatment, but overly technical explanations were not preferred, as they could negatively affect the therapeutic relationship and hinder effective communication [18,21,24,33].

The communication and behavior of physical therapists are also influenced by cultural diversity. With increasing population diversity, promoting cultural competence in rehabilitation is a critical concern [34]. Differences in cultural backgrounds may increase the risk of miscommunication and cultural barriers [35]. Verbal and non-verbal communication vary across cultures, including differences in speech volume, personal space, eye contact, and body language [36]. For example, norms around eye contact, personal space, and physical touch differ across cultures and can influence clinical communication [37]. In the Saudi context, culturally specific expectations regarding modesty and gender-appropriate interaction are especially relevant, underscoring the importance of cultural competence for respectful and effective physical therapy practice in this setting [38].

Despite the importance of bedside manners, research on how they affect physical therapy practice in Saudi Arabia remains limited in both qualitative and quantitative aspects. Recent research in Saudi Arabia has examined therapists’ knowledge and beliefs regarding vestibular rehabilitation [39] and the cultural and social determinants of rehabilitation [38]; however, bedside manners in physical therapy remain unexplored in this context. Addressing this gap is essential to enhance therapeutic relationships, support ethical decision-making, and improve patient-centered care in physical therapy practice in Saudi Arabia. Therefore, this study aims to explore therapists’ opinions and perceptions regarding bedside manners and ethics in physical therapy practice in Saudi Arabia, contributing to a better understanding of factors influencing patient–therapist interaction in this context. The bedside manner domains examined were drawn from the research team’s clinical experience and the existing literature.

## 2. Materials and Methods

### 2.1. Design

This study employed a cross-sectional design using a mixed-methods approach, integrating both quantitative and qualitative data. Quantitative data were collected through surveys to obtain numerical evidence regarding physical therapists’ knowledge and perspectives on bedside manners, while qualitative data was gathered through interviews to explore in-depth experiences and attitudes of therapists. This combination provided a comprehensive understanding of their perspectives and allowed the quantitative and qualitative findings to complement one another [40].

### 2.2. Study Participants, Recruitment and Sampling

A convenience sample of 147 physical therapists was recruited from various sites to ascertain the diversity of participants. A convenience sample was chosen due to the exploratory nature of the study, practicality, and participant accessibility. Participants included in this study were licensed Saudi physical therapists practicing in either the public or private sector except for a small number of participants (10; 6.8%) where they were licensed but not employed. From this sample, 22 participants volunteered to participate in the qualitative interview phase and were subsequently interviewed. No purposive selection criteria were applied beyond the study eligibility requirements. The interview sample included participants from a range of clinical and workplace settings, allowing for the exploration of diverse experiences and perspectives (Supplementary Table S1). Moreover, 22 participants were considered appropriate for the qualitative design and provided sufficient in-depth data to address the study objectives [41]. All volunteers who consented to participate were interviewed, and thematic saturation was deemed to have been achieved during analysis, as no new themes emerged from the final interviews. The study was conducted over six months, from June 2024 to November 2024, across multiple hospitals in different regions of the Kingdom of Saudi Arabia. Participants were recruited through advertisements, word of mouth, and outreach to local and national physical therapy departments and centers. Recruitment materials indicated that a research study was being conducted and sought the involvement of physical therapists. Out of approximately 172 physical therapists, 147 agreed to participate and completed the survey (response rate approximately 85%). Ethical approval was obtained from the Institutional Review Board at Qassim University (IRB 24-91-06 and 20 May 2024), and informed consent was secured from all participants prior to their involvement.

### 2.3. Data Collection

A mixed-methods design was employed, comprising quantitative and qualitative components.

Quantitative:

A 28-item self-administered questionnaire was developed, consisting of two segments. The first segment (8 items) collected demographic information: age, gender, academic training, employment status, years of experience, work setting, type of setting, and physical therapy specialty. The second segment (20 items) assessed perceptions of bedside manners, divided into 10 yes–no questions (section A) and 10 items rated on a 5-point Likert scale (section B). The questionnaire was available in paper-based and online formats (Google Forms) to maximize accessibility. The questionnaire content was generated by the research team through expert clinical consensus and informed by the existing bedside-manners literature.

The draft questionnaire underwent content validation before data collection. Eight physical therapy faculty members with more than five years of clinical experience independently rated the relevance of each item using Lynn’s four-point relevance scale: 1 = not relevant, 2 = somewhat relevant, 3 = quite relevant, and 4 = highly relevant. Item-level content validity indices (I-CVIs) were calculated as the proportion of experts assigning a rating of 3 or 4 to each item. The I-CVI values ranged from 0.88 to 1.00, and the scale-level content validity index, calculated as the average I-CVI across items (S-CVI/Ave), was 0.90. An I-CVI of 0.78 or greater was considered acceptable. Although all items met this criterion, expert feedback identified opportunities to improve wording, clarity, and cultural appropriateness. Accordingly, Section A item 8 and Section B item 7 were revised. In addition, Section B item 10 was added based on expert consensus. No items were removed. A separate pilot or pre-test of the questionnaire was not conducted prior to data collection.

The survey evaluated domains relevant to therapeutic interactions, patient outcomes, therapeutic alliance, and service delivery. For example, one item assessed therapists’ recognition of the importance of first impressions for treatment success. Completion took approximately 5–7 min, with participants informed of their right to withdraw. Responses were securely stored, and unique participant codes ensured confidentiality. The survey was not anonymous at the point of collection: each data collector knew the identity of the participants they personally recruited, but not those recruited by other collectors. Each collector then de-identified their own participants by assigning unique codes, so that the compiled dataset contained no direct personal identifiers. The faculty members did not know the identity of all the participants.

Qualitative:

Qualitative data were collected through semi-structured interviews conducted either by telephone or face-to-face, according to participant preference and logistical considerations, and audio-recorded with participants’ informed consent. Although interview mode may have influenced participant responses and the depth of interaction, the use of a standardized interview guide across both formats helped maintain consistency in questioning and minimize variation in data collection [42]. A total of 22 interviews were conducted by nine trained researchers (M.M.A., S.H.A., R.M.A., J.B.A., W.A.A., F.A.A., R.A., R.K.A., F.A.) under the supervision of a faculty member (S.M.A.). Prior to data collection, interviewers received guidance from the faculty supervisor regarding the study objectives, interview procedures, ethical considerations, and use of the interview guide. Open-ended questions enabled participants to freely express their perceptions and experiences, while follow-up questions were used to explore emerging issues in greater depth [43]. Interviews lasted between 15 and 45 min, and participants were given the option to conduct the interview in either Arabic or English; however, all participants chose Arabic. Researchers emphasized voluntary participation, confidentiality, and the absence of any consequences related to participation or non-participation throughout the interview process.

Audio recordings were transcribed verbatim and subsequently translated into English by the researchers who conducted the interviews. Translation accuracy was reviewed through comparison with the original Arabic transcripts and supported by language-checking software. No formal back-translation procedure was undertaken. To enhance data credibility, member checking was conducted where participants were provided with their transcripts to verify the accuracy of the recorded information [44]. To protect participant confidentiality, each participant was assigned a unique identifier. A combination of Roman numerals (i–xi) and numerical codes (1–147) was used, whereby the Roman numeral denoted the interviewer, and the numerical code denoted the participant. These identifiers were used consistently throughout transcription, analysis, and reporting of the findings. The same coding system was maintained across qualitative and quantitative datasets to facilitate comparison while preserving participant anonymity. Following analysis, all recordings and transcripts were stored securely according to the study’s data management procedures.

Interviewers and interviewees were drawn from a range of institutions, work settings, and experience levels; not all interviewers were senior to the participants, several participants were more experienced than their interviewer, and most did not share a work environment. Importantly, no interviewee was supervised or mentored by their interviewer, reducing the likelihood of a coercive disclosure context. This was verified by the supervising faculty members, who confirmed with each interviewer that none of their assigned interviewees were under that interviewer’s clinical supervision or mentorship.

### 2.4. Data Analysis

Categorical data were summarized using frequencies and percentages, and continuous data using means and standard deviations. Among the demographic variables collected, gender, work setting, and years of experience were chosen as the factors of interest for examining differences in bedside-manner perceptions: gender because prior evidence links practitioner gender to communication and interpersonal behaviors [45,46,47,48]; work setting because settings differ in patient acuity, case mix, and staffing, which may shape behaviors such as therapist presence and attention to case severity; and years of experience because the interpersonal and ethical aspects of care may evolve over a clinician’s career. Responses were summarized overall and by these factors; differences by gender are also illustrated.

Responses were measured on a 5-point scale, ranging from “strongly agree” to “strongly disagree.” For descriptive summary, responses of “strongly agree” and “agree” were combined into a single “agree” category, while “strongly disagree” and “disagree” were combined into a “disagree” category. Responses marked as “neutral” were kept as a separate category, resulting in three levels (agree/neutral/disagree) to summarize responses, in addition to the original five-point distribution.

The qualitative data were analyzed using Braun and Clarke’s six-phase thematic analysis approach, which involved familiarization with the data through repeated reading of transcripts, generation of initial codes, searching for potential themes, reviewing and refining themes, defining and naming themes, and producing the final report [49]. The four researchers (S.H.A., R.M.A., J.B.A., and W.A.A.) independently coded the transcripts. Coding was performed on the English translations, with the original Arabic transcripts consulted throughout to preserve meaning. M.M.A. then worked with the team to compare the initial codes, discuss differences in interpretation, and agree on the coding framework. The agreed coding scheme was subsequently reviewed with the supervising faculty member (S.M.A.) to enhance analytical rigor. Formal inter-coder agreement was not calculated; instead, coding decisions were developed through collaborative discussion and interpretation among the research team, consistent with Braun and Clarke’s approach to thematic analysis. Themes were then reviewed, refined, and organized in relation to the research questions and existing literature, resulting in the identification of seven final themes.

Several strategies were employed to enhance the credibility, dependability, and transparency of the analysis. Preliminary themes were shared with participants as members checking for review, clarification, and feedback, enabling participants to comment on the researchers’ interpretations and contributing to the credibility of the findings [44]. Peer debriefing was undertaken throughout the research process with the faculty supervisor (S.M.A.), who provided critical feedback and challenged interpretations where appropriate [50]. In addition, an audit trail was maintained documenting coding decisions, theme development, supporting data extracts, and modifications made during the analytical process [51]. These procedures enhanced the trustworthiness of the findings and provided transparency regarding the progression from raw data to final themes.

## 3. Results

**Figure 1:**
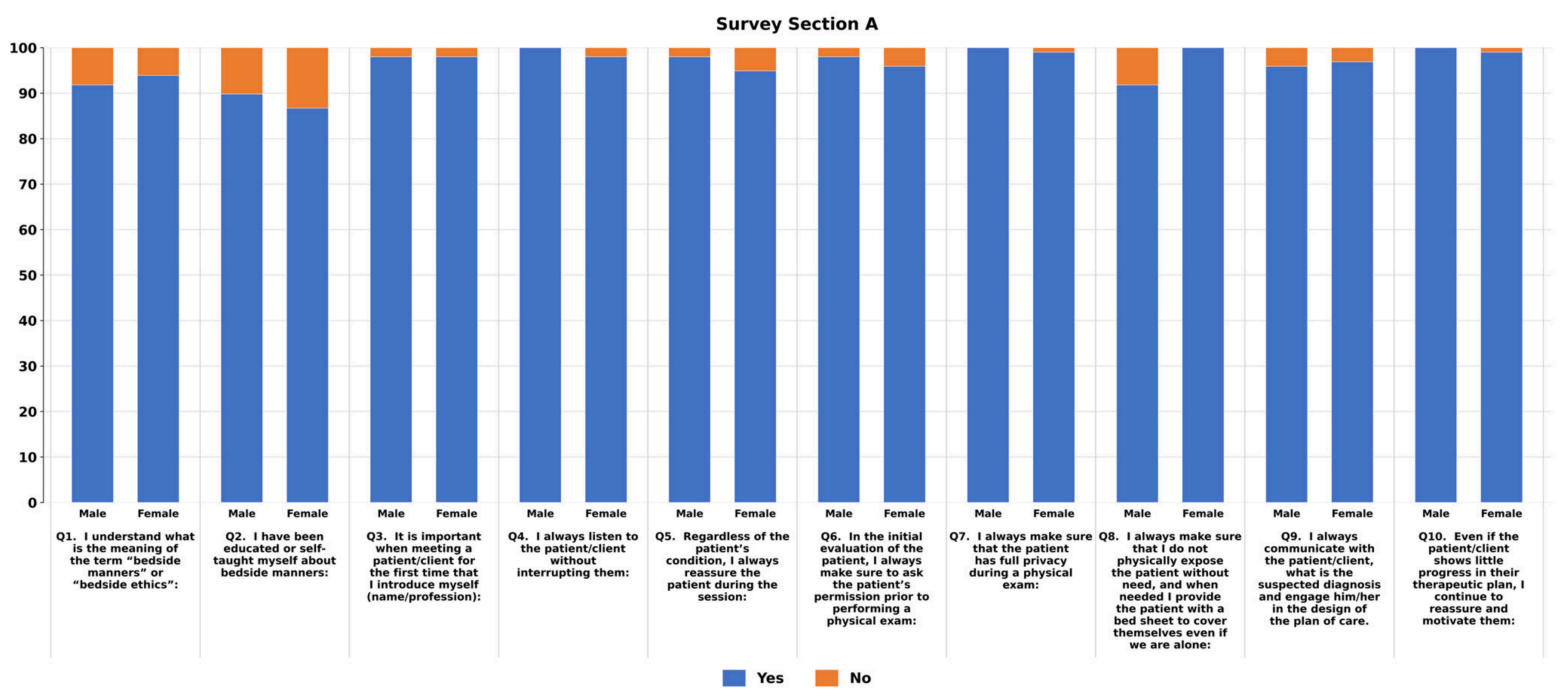
Percentage of male and female physical therapists responding “Yes” or “No” to questions regarding bedside manners practices and awareness.

**Figure 2:**
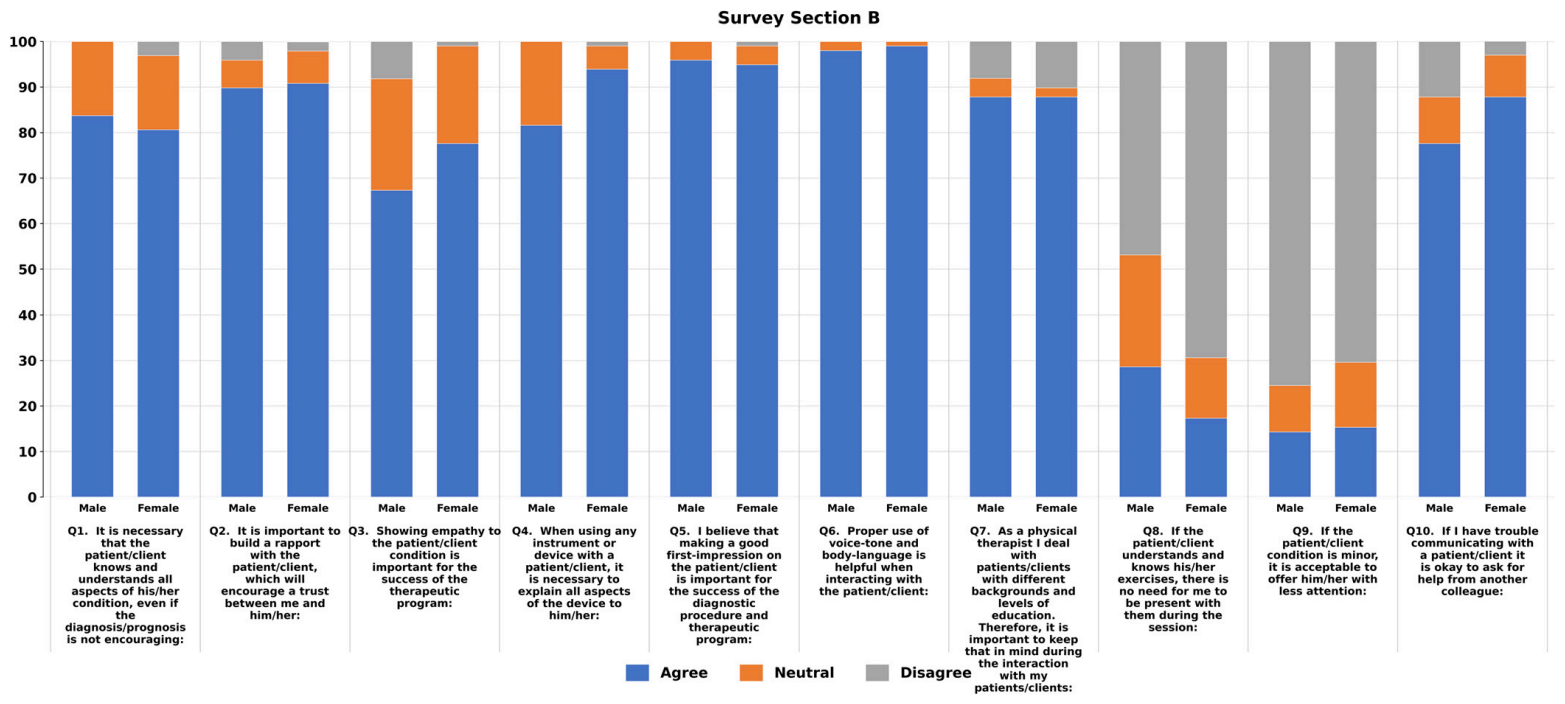
Distribution of male and female physical therapist responses to statements regarding bedside manners (survey section B), presented as percentage agreement (agree/strongly agree), neutrality, and disagreement (disagree/strongly disagree).

A total of 147 participants were enrolled in the study. All participants completed the quantitative component, and only 22 took part in the qualitative interviews. The majority of participants were female (66.7%) and between 20–30 years of age (70.7%). The largest group was employed in government hospitals (44.2%), and most worked in outpatient departments. Detailed demographic characteristics are presented in Table 1. Result from the first part of the survey indicated that “yes” responses constituted the highest percentages across the yes/no questions, as shown in Table 2. In the second part of the survey (Likert-scale items), responses are summarized in Table 3; most items showed high agreement, while items on therapist presence (Question 8) and attention to minor cases (Question 9) showed greater variability. Subgroup patterns by gender, work setting, and years of experience are described below. Consistent with the findings of the first and second part of the survey, Figure 1 and Figure 2 illustrate differences in response proportions between female and male physical therapists.

(a) Quantitative Section
  (A) Section A (yes/no items)
    In Section A, agreement was high across all ten yes/no items, ranging from 87.8% to 99.3% “yes” (Table 2). Endorsement was highest for ensuring the patient’s full privacy during physical examination (Question 7) and for continuing to reassure patients who show little progress (Question 10), both 99.3%, followed by listening without interrupting (Question 4, 98.6%) and introducing oneself at the first meeting (Question 3, 98.0%). The lowest, though still high, were having been educated or self-taught about bedside manners (Question 2, 87.8%) and understanding the term “bedside manners” (Question 1, 93.2%). Privacy-related conduct was also strongly endorsed, including not physically exposing the patient unnecessarily (Question 8, 97.3%). These uniformly high proportions indicate a ceiling effect across Section A.
  (B) Section B (Likert items)
    In Section B, most statements drew strong agreement (Table 3). Agreement was highest for using appropriate voice tone and body language (Question 6, 98.6%), making a good first impression (Question 5, 95.2%), and building rapport and trust with the patient (Question 2, 90.5%). Two items were worded so that disagreement reflects recommended practice, and showed the opposite pattern: most therapists disagreed that there is no need to remain present once a patient knows their exercises (Question 8; 61.9% disagreed vs. 21.1% agreed) and that minor cases may receive less attention (Question 9; 72.1% disagreed vs. 15.0% agreed), indicating strong support for therapist presence and for equitable attention regardless of case severity. Neutral responses were most frequent for the importance of showing empathy (Question 3, 22.4%) and for ensuring the patient understands all aspects of their condition (Question 1, 16.3%).
  (C) Differences by gender
    Responses were broadly similar between female and male therapists across most items. A few items differed. For the Section A privacy item (Question 8), all female therapists (100%) answered “yes,” compared with 91.8% of male therapists (four male respondents answered “no”). In Section B, a higher proportion of female than male therapists agreed that all aspects of a device should be explained to the patient (Question 4; 93.9% [92/98] vs. 81.6% [40/49]), and a higher proportion of female therapists disagreed that there is no need to remain present once the patient knows their exercises (Question 8; 69.4% [68/98] vs. 46.9% [23/49]). These differences are illustrated in Figure 1 and Figure 2.
  (D) Differences by work setting
    Responses were broadly similar across work settings on most items; two items in Section B showed variation. For Question 8 (therapist presence), disagreement with leaving patients alone was highest among therapists in private clinics (70.6%) and government hospitals (66.2%), and lowest among those in rehabilitation hospitals (41.2%), who were correspondingly the most likely to agree (47.1%) when patients were familiar with their treatment plans.
    For Question 9 (giving minor cases less attention), disagreement was highest among therapists in government hospitals (78.5%) and private clinics (76.5%). The single Community Care respondent (n = 1) is reported but, given the single respondent, is not included in the between-setting comparison. The full Q8 and Q9 cross-tabulations across all five work settings are provided in Supplementary Table S2.
  (E) Differences by experience
    Responses were broadly similar across experience levels. Disagreement with leaving patients alone once they know their exercises (Question 8) was somewhat lower among more experienced therapists (0–5 years, 67% [69/103]; 5–10 years, 53% [17/32]; >10 years, 42% [5/12]), with correspondingly more neutral responses. The agreement with showing empathy is important (Question 3) was slightly lower in the most experienced group (58% [7/12] versus approximately 76% among earlier-career therapists). Endorsement of privacy and most other items was high across all experience levels. The most experienced group was small (n = 12); therefore, these patterns should be interpreted with caution.
(b) Qualitative analysis:
  (A) Building trust
    Therapists consider bedside manners essential in their practice, as they are crucial for building trust between the therapist and the patient, as well as for the fundamentals of the profession. Participants described trust as developing through behaviors that demonstrate respect and attentiveness to patients’ concerns. Active listening and clear communication were perceived as helping patients feel heard and informed, while maintaining privacy and professional conduct were viewed as reinforcing confidence in the therapist and the therapeutic relationship.
    Therapist vi12: “Bedside ethics are important in order to build trust between us and the patients, and this contributes to the success of the treatment plan.”
    Therapist ii6: “patients need someone who listens to them and allows them to feel comfortable speaking and expressing what they have to say.”
  (B) Positive contribution to care
    The majority of participants described the perceived value of bedside manners as operating through the development of trust. In their accounts, trust was viewed as encouraging patients to communicate openly, consider therapists’ recommendations, and engage more actively in the therapeutic process.
    Therapist xi2: “Certainly, it has an impact. It’s one of the things that make the patient adhere to the treatment plan, consider your decisions, and value your opinion. If you maintain these aspects, all these ethical practices will enhance the trust between you and the patient.”
    However, these perceptions were not shared by all participants. One participant expressed uncertainty “Maybe not sure” (v1), while another stated “From a technical standpoint, it doesn’t make a difference” (x26). Although differences in work setting appeared to coincide with variation in participants’ views, work setting was not examined as a determinant in this study. Differences in work settings appeared to coincide with variation in participants’ perceptions. In this theme, the participant working in an inpatient setting described bedside manners as important for fostering trust and engagement, whereas the participant working across inpatient and outpatient settings expressed uncertainty, and the participant working primarily in an outpatient setting questioned their influence. Although this pattern emerged from participants’ accounts, work setting was not directly examined as a determining factor in the present study and should therefore be considered only as an explanation for the variation in views.
  (C) Body language
    Therapists viewed effective non-verbal communication as beneficial for establishing rapport and supporting patient comfort. They find it effective in containing and supporting the patient, which in turn provides therapists with the patient’s attention and commitment.
    Therapist ii6: “yes of course, body language is very important when dealing with patients, the patients always recognize therapist body language. One of the patients complained about the doctor body position and how he was standing while he takes her history which make her anxious.”
    Therapist ii1: “it affects the quality of the session, and how open the patient is to the therapy. The patient will determine whether or not to continue attending after the initial session.”
    Non-verbal communication, including eye contact, facial expression, posture, tone of the voice, therapeutic touch, and respectful positioning, plays an important role in physical therapy practice. These behaviors can enhance patient comfort, promote trust, support privacy and dignity, and help maintain appropriate professional boundaries. These qualities were viewed as contributing to positive therapeutic interactions and patient-centered care.
  (D) Differing views on empathy
    When the interviewer asked the therapists about empathy, and whether it was important during treatment, participants’ views fell into three broad perspectives. Unlike bedside manners, which participants generally described as observable professional behaviors and communication practices, empathy was discussed in relation to understanding patients’ experiences and emotional needs.
    The first part agreed to use it during sessions.
    Therapist vi11: “Yes, of course, I must show my understanding of the pain they feels and show my full empathy for them.”
    The second did not prefer to use it during sessions.
    Therapist vi12: “No, sometimes empathy may not be necessary. What is important is to provide care to the fullest extent and leave me satisfied.”
    Finally, the third opinion emphasized that empathy should be applied according to the patient’s personality. They indicated that empathy may not be appropriate in all situations and that its use should be adapted to individual patient characteristics and clinical circumstances.
    Therapist ii6: “Perhaps empathy would take another path if it existed. Empathy is not always the right thing, and you can use it according to the patient’s health condition and personality. Some patients will be affected by empathy.”
    The differing opinions suggest that therapists do not view empathy as a universally beneficial approach. Instead, they may tailor its use based on individual patient characteristics and clinical circumstances, reflecting the need to balance emotional involvement with therapeutic effectiveness and professional objectivity. These differing views may also reflect variation in therapists’ work settings or clinical responsibilities; however, these factors were not directly examined and this interpretation remains speculative. Another possible explanation may relate to differences in work settings and clinical responsibilities. For example, therapists working in inpatient settings may encounter patients with greater emotional and psychological needs, whereas those in outpatient settings may place greater emphasis on maintaining professional boundaries and encouraging patient independence. However, these factors were not directly examined in the present study.
  (E) Therapist presence
    Some therapists agreed on the importance of the therapist’s presence throughout the session, while others disagreed.
    Therapist iv13: “It is important that there is monitoring of the patient, even if the sessions are consecutive and the patient masters performing the therapeutic exercises. It is important that there is supervision to ensure the patient’s safety first, and also to monitor the patient’s performance in their treatment program.”
    Therapist x20 “If I’ve been treating them for a long time, I don’t mind leaving them because they know their exercises and everything they need. But it’s not about just leaving them completely; I need to check on them regularly, so they feel that I care about them.”
    Participants expressed differing views regarding the extent to which therapists should remain continuously present during treatment sessions. Continuous therapist presence was perceived as particularly important in situations involving patient safety, close supervision, complex interventions, or when reassurance and emotional support were needed. In contrast, participants considered brief therapist absence acceptable during low-risk activities when patients were stable, adequately instructed, and able to perform tasks independently, provided that appropriate monitoring and supervision were maintained.
  (F) Patient equity
    In clinical practice, therapists emphasize the importance of delivering fair and equal care to all patients, regardless of age, diagnosis, or the level of difficulty involved in the condition. They make sure that they treat them equally and give them their needed time.
    Therapist iv11: “as long as the patient is concerned and comes just to solve the problem even if it is simple, I give the same attention as any other patients whether it is a difficult or temporary case.”
    Other therapists have another opinion. They of course will treat all patients equally, but some cases need more time like geriatric patients or complicated cases that need a multidisciplinary team.
    Therapist xi1: “of course the level of attention is the same but there is a difference in cases but not the attention. The difference is in my time with the patients … the main attention that in the beginning that I hear and understand for sure will be equal. But if their problem is complicated of course, I hear them more, I sit with them more and communicate more because the case will have more referrals since the problem has more complex details.”
    Participants generally described patient equity as providing the same level of respect, attention, and professional commitment to all patients regardless of age, diagnosis, or case complexity. They distinguished between equality of attention and equality of time allocation, noting that patients with more complex conditions may require additional communication, assessment, or multidisciplinary coordination to meet their clinical needs. These findings suggest that participants perceived equitable care as adapting support to patient needs while maintaining fairness and respect across all patients. However, these findings reflect therapists’ perceptions of equitable practice and should not be interpreted as an objective assessment of equity within clinical practice.
  (G) Seeking help from colleagues
    Most of the therapists agreed to ask their colleagues if they are facing any issues with the patient’s case, but this should be away from the patient or after the session.
    Therapist x2: “It depends on the situation. If I encounter a new case and my colleague has sufficient experience in that, I will maintain the level of trust between me and the patient and I will consult my colleague later on.”
    Participants viewed seeking support from colleagues as an aspect of professional bedside manners and ethical practice. Recognizing situations that exceeded one’s expertise and consulting more experienced colleagues when necessary was perceived as reflecting professional responsibility, awareness of professional limitations, and a commitment to maintaining patient trust and professional credibility. Participants also perceived collaboration with colleagues as a way of supporting appropriate patient care, particularly when managing unfamiliar or complex cases, while helping maintain professional boundaries and promote patient safety.
    Additional quotations from the thematic analysis are summarized in Table 4.

## 4. Discussion

This study is, to our knowledge, the first to explore perceptions of bedside manners among physical therapists in Saudi Arabia using a mixed-methods approach. The quantitative findings identified several domains, including patient privacy, explanation of therapeutic tools, therapist presence, attention to minor cases, and empathy. Perceptions in some of these domains differed across gender and work setting. The qualitative interviews generated seven themes (building trust, perceived contribution to care, body language, differing views on empathy, therapist presence, patient equity, and seeking help from colleagues). Together, these two components provide a broader understanding of how physical therapists conceptualize bedside manners and the contextual factors shaping these perceptions. Notably, empathy elicited divergent perspectives among participants.

Maintaining patient privacy is essential in physical therapy practice in Saudi Arabia, yet the literature on this aspect remains limited. In the descriptive data, all female therapists emphasized preserving patients’ physical privacy and providing a bed sheet even when patients were alone, compared with 91.8% of male therapists; because this difference rests on only four male “no” responses, it should be interpreted with caution. This emphasis on privacy, safety, and confidentiality is consistent with prior descriptions of ethical physiotherapy practice [52], and in the Saudi context it may be reinforced by the gender-segregated structure of physical therapy education and the largely same-sex nature of clinical care [38,53]. Whether such gender-related differences also arise where training and care are mixed-sex, as in the United States and many European countries, remains an open question for future cross-cultural research.

Most therapists perceived explaining instruments or devices used during treatment as an integral part of ethical and effective clinical practice, reflecting a commitment to enhancing patient understanding and informed participation in the therapeutic process. This finding is consistent with previous studies emphasizing the therapist’s ability to provide clear and simple explanations of conditions and treatment procedures as an essential professional skill [18,19,21,23,24,25,26,33,54]. Patients tend to value healthcare practitioners who provide detailed explanations about assessment and treatment procedures during sessions [29]. Greater agreement was observed among female therapists compared to males regarding the necessity of explaining devices and equipment during therapeutic sessions (93.9% vs. 81.6%). Previous studies on physician communication suggest that female practitioners often share more information, provide greater acknowledgment, and engage more in collaborative and positive interactions [44,45,46,47].

Maintaining the therapist’s presence throughout treatment sessions enables effective guidance, monitoring of patient safety, and proper execution of exercises. However, therapists may allow patients to continue exercises independently when they are familiar with the treatment, provided regular monitoring is maintained, as illustrated by therapist x20: “If I’ve been treating them for a long time, I don’t mind leaving them because they know their exercises and everything they need. But it’s not about just leaving them completely; I need to check on them regularly, so they feel that I care about them.”. Therapists also emphasized the ethical importance of seeking patient consent before leaving, ensuring that any absence is brief and work-related, as noted by therapist xi2:“…In the end, you would ask the patient’s permission before leaving them, and most likely, you won’t leave them for a long time or for any purpose other than the work itself”.

Responses on therapist presence varied by work setting and gender. Disagreement with leaving patients alone was highest among therapists in private clinics (70.6%) and government hospitals (66.2%), whereas therapists in rehabilitation hospitals were the most likely to agree (47.1%) when patients were familiar with their treatment plans. One possible explanation, which the present data cannot test, is that heavy workloads and limited staff availability in rehabilitation settings contribute to this tendency. Previous research in rehabilitation nursing shows that high workload can negatively affect quality of care and increase the risk of adverse events such as falls and infections [55,56,57]. Gender differences were also observed, with female therapists placing greater importance on remaining with patients during therapy sessions compared to male therapists (69.4% vs. 46.9%). This pattern is consistent with evidence that female clinicians tend to engage in more patient-centered communication and longer, more interpersonally oriented consultations than their male counterparts [58]. Despite its importance, few studies address therapist presence during physical therapy sessions, highlighting the need for further research.

The results indicate that physical therapists demonstrate a strong professional attitude by ensuring comprehensive healthcare delivery for all patients, regardless of case severity. Achieving recovery while valuing patients as individuals reflects a balance between clinical expertise and a person-centered approach [59,60,61]. Understanding and implementing principles of health justice is essential within physical therapy practice [62,63]. Across work settings, most therapists disagreed with providing less attention to minor cases (government hospitals 78.5%, private hospitals 66.7%, private clinics 76.5%, and rehabilitation hospitals 47.1%, alongside the single community-care respondent). In rehabilitation hospitals, fewer than half of the therapists disagreed (47.1%), with an equal proportion in agreement. Although simple cases may be perceived as requiring less attention, therapists emphasized the importance of providing appropriate care for every patient, as illustrated by participant vi12: “Yes, there may be less attention paid to simple cases, but we must remain careful to provide appropriate care for each case”. Additionally, variations in services were attributed to individual clinical needs rather than unequal care, as noted by participant v1, “Attention is the same for everyone; service may vary depending on the situation.” These findings reflect strong professional responsibility and commitment to patient-centered care, emphasizing that case severity should not compromise care quality. Ensuring equity in patient care represents an ethical obligation and aligns with Saudi Arabia’s Vision 2030 objective of promoting a prosperous and fulfilling life for all citizens [64].

Empathy is widely recognized as a fundamental component of effective therapeutic communication. However, the findings revealed variability among physiotherapists in its application: some consistently endorsed empathy, others avoided it, and a third group adjusted their approach according to the patient’s personality. Several therapists emphasized maintaining a balanced approach to patient care, as illustrated by physiotherapist iv11: “No excess or negligence. We must show that we empathize with them, but at the same time, let the emotion not prevail and make us be too lenient with patients”. Evidence indicates that healthcare practitioners’ empathy and compassion may be influenced by factors such as the patient’s rehabilitation stage, degree of impairment, practitioner gender, years of experience, and treatment environment [65]. A Korean survey found that rehabilitation outpatients who perceived physicians as highly empathic reported higher satisfaction and better adherence to treatment [66]. Similarly, a cross-sectional study in Germany identified perceived physician empathy as a significant predictor of patient satisfaction and treatment acceptance, although it did not significantly affect cooperation or adherence [67]. To our knowledge, no published study has specifically examined empathy among physiotherapists in Saudi Arabia. Given the observed variability, further research is needed to explore the cultural, educational, and organizational factors shaping therapists’ empathic behavior and its impact on patient outcomes in rehabilitation settings.

Despite the element-specific differences observed, the findings demonstrated a high level of agreement between male and female therapists regarding bedside manners. Over 90% of participants in both groups reported positive perceptions across most items, with highly consistent responses in key aspects of professional conduct and patient-centered care, including communication, consent, reassurance, motivation, and collaboration with colleagues. These results indicate broad similarity between male and female therapists in overall perceptions of bedside manners and suggest that therapists share a common professional standard and approach to patient-centered care.

## 5. Strengths and Limitations

One of the key strengths of this study is the diversity of the sample. Participants were drawn from various hospitals (government and private) and multiple urban regions across the Kingdom, enhancing the contextual depth of the findings. This geographic and institutional variation provides a clearer understanding of how different environments may relate to the ethical practice and decision-making of physical therapy professionals. The mixed-methods design allowed the survey findings to be complemented by richer qualitative accounts, and the study addresses bedside manners in physical therapy, a topic that remains under-researched, particularly in Saudi Arabia.

Several limitations should be acknowledged. The study used convenience sampling, and recruitment through advertisements and word of mouth for a survey explicitly about bedside manners. This may have attracted therapists who already value these behaviors, introducing selection bias. The findings should therefore not be generalized beyond Saudi physical therapists who volunteered for such a survey, and external validity is limited despite the diversity of settings sampled. There was a marked gender imbalance, with fewer male than female participants, which constrains comparisons between groups, and the sample was weighted toward younger, early-career therapists (70.7% aged 20–30; 70.1% with 0–5 years of experience), which may limit transferability to more experienced practitioners.

In addition, most survey items showed very high agreement (ceiling effects), leaving little variability and raising the possibility of social desirability bias. While the questionnaire’s content was validated, the ceiling effects and potential social-desirability bias noted above are still possible. The study relied on self-reported perceptions, which may not reflect actual bedside behavior, and did not include patient-reported outcomes. For the qualitative component, no formal back-translation of the interview material was undertaken. Finally, the scarcity of research specifically examining physical therapists’ perspectives on bedside manners made it challenging to position the findings within a broader theoretical framework, and expanding discipline-specific research would help build a stronger evidence base. In addition, nearly one-quarter of participants (23.1%) trained at the corresponding author’s institution, which may limit the diversity of the sample. Also, the intercoder agreement process of the qualitative component was not conducted. While this may limit the formal dependability and reliability of the coding process, the research team mitigated this by holding extensive, iterative debriefing sessions to discuss, refine, and reach a consensus on the final themes. This ensured that the conceptual framework was thoroughly debated and agreed upon by multiple researchers.

## 6. Conclusions

In conclusion, this study explored Saudi physical therapists’ perceptions of bedside manners and identified key components of professional communication and clinical interaction. The findings indicated general agreement on the importance of communication, consent, reassurance, motivation, and collaboration with colleagues, all of which therapists reported as important for patient comfort, adherence, and treatment acceptance. Some variation related to gender and work setting was also observed. Research focusing specifically on physical therapists’ practice in Saudi Arabia remains limited, highlighting the need for further studies across diverse clinical contexts.

## Figures and Tables

**Table 1:** Characteristics of therapists who participated in the study.

Demographic Characteristics	(n = 147)
Age, years	20–30—104 (70.7%) 31–40—38 (25.8%) 41–50—5 (3.4%)
Sex	Female—98 (66.7%) Male—49 (33.3%)
Academic training	Qassim University—34 (23.1%) King Saud University—20 (13.6%) Umm Al-Qura University—4 (2.7%) King Khaled University—5 (3.4%) King Abdulaziz University—12 (8.2%) University of Hail—9 (6.1%) Taif University—4 (2.7%) Jazan University—10 (6.8%) Najran University—1 (0.7%) Imam Abdurahman Al Faisal University—13 (8.8%) Prince Sattam University—4 (2.7%) Shaqra University—2 (1.4%) Majmaah University—12 (8.2%) Prince Noura University—8 (5.4%) Other—9 (6.1%)
Employed	Yes—137 (93.2%) No—10 (6.8%)
Experience, Years	0–5 years—103 (70.1%) 5–10 years—32 (21.8%) >10 years—12 (8.1%)
Work Setting	General Hospital (Government)—65 (44.2%) General Hospital (Private)—30 (20.4%) Rehabilitation Hospital—17 (11.6%) Community Care—1 (0.7%) Private Clinic—34 (23.1%)
Type of work setting	Outpatient—77 (52.4%) Inpatient—12 (8.2%) Mix (in- and out-patient)—58 (39.5%)
PT Specialty	General PT Practice—74 (50.3%) Neurology—14 (9.5%) Orthopedics/Sports—41 (27.9%) Cardiopulmonary—1 (0.7%) Geriatrics—3 (2%) Pediatrics—7 (4.8%) Home Health Care—3 (2%) Women’s Health—4 (2.7%)

**Table 2:** Questions and responses to Section A survey.

	Yes	No
1—I understand what is the meaning of the term “bedside manners” or “bedside ethics”:	137 (93.2%)	10 (6.8%)
2—I have been educated or self-taught myself about bedside manners:	129 (87.8%)	18 (12.2%)
3—It is important when meeting a patient/client for the first time that I introduce myself (name/profession):	144 (98%)	3 (2%)
4—I always listen to the patient/client without interrupting them:	145 (98.6%)	2 (1.4%)
5—Regardless of the patient’s condition, I always reassure the patient during the session:	141 (95.9%)	6 (4.1%)
6—In the initial evaluation of the patient, I always make sure to ask the patient’s permission prior to performing a physical exam:	142 (96.6%)	5 (3.4%)
7—I always make sure that the patient has full privacy during a physical exam:	146 (99.3%)	1 (0.7%)
8—I always make sure that I do not physically expose the patient without need, and when needed I provide the patient with a bed sheet to cover themselves even if we are alone:	143 (97.3%)	4 (2.7%)
9—I always communicate with the patient/client, what is the suspected diagnosis and engage him/her in the design of the plan of care.	142 (96.6%)	5 (3.4%)
10—Even if the patient/client shows little progress in their therapeutic plan, I continue to reassure and motivate them:	146 (99.3%)	1 (0.7%)

**Table 3:** Questions and responses to Section B survey.

	Strongly Agree	Agree	Neutral	Disagree	Strongly Disagree
1—It is necessary that the patient/client knows and understands all aspects of his/her condition, even if the diagnosis/prognosis is not encouraging:	69 (46.9%)	51 (34.7%)	24 (16.3%)	3 (2.0%)	0 (0.0%)
2—It is important to build a rapport with the patient/client, which will encourage a trust between me and him/her:	90 (61.2%)	43 (29.3%)	10 (6.8%)	4 (2.7%)	0 (0.0%)
3—Showing empathy to the patient/client condition is important for the success of the therapeutic program:	52 (35.4%)	57 (38.8%)	33 (22.4%)	4 (2.7%)	1 (0.7%)
4—When using any instrument or device with a patient/client, it is necessary to explain all aspects of the device to him/her:	94 (63.9%)	38 (25.9%)	14 (9.5%)	1 (0.7%)	0 (0.0%)
5—I believe that making a good first-impression on the patient/client is important for the success of the diagnostic procedure and therapeutic program:	93 (63.3%)	47 (32.0%)	6 (4.1%)	1 (0.7%)	0 (0.0%)
6—Proper use of voice-tone and body-language is helpful when interacting with the patient/client:	104 (70.7%)	41 (27.9%)	2 (1.4%)	0 (0.0%)	0 (0.0%)
7—As a physical therapist I deal with patients/clients with different backgrounds and levels of education. Therefore, it is important to keep that in mind during the interaction with my patients/clients:	101 (68.7%)	28 (19.0%)	4 (2.7%)	3 (2.0%)	11 (7.5%)
8—If the patient/client understands and knows his/her exercises, there is no need for me to be present with them during the session:	10 (6.8%)	21 (14.3%)	25 (17.0%)	57 (38.8%)	34 (23.1%)
9—If the patient/client condition is minor, it is acceptable to offer him/her with less attention:	8 (5.4%)	14 (9.5%)	19 (12.9%)	60 (40.8%)	46 (31.3%)
10—If I have trouble communicating with a patient/client it is okay to ask for help from another colleague:	62 (42.2%)	62 (42.2%)	14 (9.5%)	5 (3.4%)	4 (2.7%)

**Table 4:** Additional quotations from the thematic analysis.

Qualitative Themes	Additional Quotations
Building trust	*-* *x7: “It is very important and one of the reasons that make the patient trust you and continue with you.”* - *x14: “These are the fundamentals.”* *-* *viii17: “It is important for several reasons. First, building trust between me and the patient.”*
Positive contribution to care	*-* *vi11: “Yes, absolutely. When the patient feels comfortable during the session and their privacy is respected, it will reflect on their commitment and, consequently, their outcome.”* *-* *iv13: “Of course, it has a significant impact because it builds trust between the therapist and the patient. Trust is a crucial factor in this matter.”* - *x2: “Of course, the psychological factor plays a big role. The mental aspect affects the treatment; if the patient feels scared or uncomfortable during the session, they’ll feel like it’s not helping, and they won’t continue or make any progress.”*
Body language	- xi1: *“Ethics include communication, which is through my choice of words and tone of voice, and also includes my good listening to the patient. Attitude and communication are essential.”* - ii1: *“It affects the quality of the session, and how open the patient is to the therapy. The patient will determine whether or not to continue attending after the initial session.”* - *x26*: *“The patient is at his weakest point and seeks help to get out of this situation. He also looks for indicators and any hope. These points are very important in addition to the fact that they affect the reputation of the specialist.”*
Differing views on empathy	*-* *v2: “What is important is understanding the patient’s condition, reassuring him, alleviating his pain, and improving his psychological state, which is important for the physical therapist”* - ii1: “Yes, but according to the personality of the patient in front of me, I will make a first impression of the patient. If I empathize with him, he will pay attention.”
Therapist presence	*-* *x7 “Almost, yes. cases require direct supervision. For athletes or those with experience, you can monitor them from a distance, but you should still be in the same space. However, for elderly individuals or those who are untrained, I must be with them to ensure their safety.”* *-* *xi2 “I believe it depends on the patient’s personality and condition. If the patient is new, it’s better not to, but with some patients, you begin to understand their personality and realize they have no issue with this matter, so it’s fine. Other patients may show you that they could be bothered or it could cause a problem. In the end, you would ask the patient’s permission before leaving them, and most likely, you won’t leave them for a long time or for any purpose other than the work itself”*
Patient equity	- v1: *“Attention is the same for everyone, service may vary depending on the situation”* - iv13: *“No, care for patients of different ages and different conditions is fair and equal, regardless of the difficulty or ease of the condition, or from a medical standpoint, the service is equal and fair for all.”*
Seeking help from colleagues	*-* *iv13: “It’s very important to exchange the knowledge between the therapists, when I take a consultation from other therapist in some cases, it’s affecting on improve the treatment plan and reach for the best outcome with patient which is the main goal”* - *x20: “It’s important even for me or any condition, the patient is not in his normal situation and has worries about his exercise’s performance if it’s correct or not and it’s may harmful”*

## Data Availability

The data presented in this study are available on request from the corresponding author due to privacy concerns, as the dataset contains potentially identifying participant information.

## Supplementary Materials

The following supporting information can be downloaded at: https://www.mdpi.com/article/10.3390/healthcare14152250/s1, Table S1. Characteristics of the 22 physical therapists who participated in the qualitative interviews. Table S2. Cross-tabulation of Section B Questions 8 and 9 by work setting (n = 147).
