# Supplementary material for: Physical Therapists’ Self-Reported Perceptions of Bedside Manners in Saudi Arabia: A Mixed-Methods Study
Source: Healthcare (Basel). 2026 Jul 23;14(15):2250. doi: 10.3390/healthcare14152250 (PMC13465485; doi:10.3390/healthcare14152250)
Supplement: Supplementary file 1 [file healthcare-14-02250-s001.zip › Supplementary Table S1 - Interview participant characteristics.pdf]

Table S1. Characteristics of the 22 physical therapists who participated in the qualitative interviews.

ID	Age	Sex	University	Experience (years)	Work setting	Setting type	PT specialty
ii1	28	F	QU	1	Private clinic	Outpatient	Orthopedics/Sports
ii6	26	F	QU	3	General hospital (private)	Mixed	General PT practice
iv11	26	F	QU	2	General hospital (private)	Mixed	General PT practice
iv13	30	F	QU	4	General hospital (government)	Outpatient	General PT practice
v1	29	F	TU	5	General hospital (government)	Mixed	Orthopedics/Sports
v2	30	F	QU	4	General hospital (government)	Outpatient	Pediatrics
vi11	28	F	JU	5	Private clinic	Outpatient	General PT practice
vi12	29	M	QU	5	Private clinic	Outpatient	General PT practice
viii17	24	M	UOH	1	Private clinic	Outpatient	General PT practice
viii18	27	F	QU	1	Private clinic	Outpatient	General PT practice
ix1	34	M	KSU	10	General hospital (government)	Inpatient	Orthopedics/Sports
ix2	31	F	QU	5	Private clinic	Outpatient	Women's health
x2	31	M	IAU	8	Private clinic	Outpatient	Orthopedics/Sports
x5	25	M	IAU	2	General hospital (private)	Mixed	General PT practice

x7	31	M	IAU	8	General hospital (government)	Mixed	Orthopedics/Sports
x13	25	F	KAU	2	Private clinic	Outpatient	General PT practice
x14	46	F	IAU	24	General hospital (government)	Mixed	Geriatrics
x15	28	F	QU	2	General hospital (private)	Mixed	General PT practice
x20	24	M	IAU	1	Private clinic	Outpatient	General PT practice
x26	26	M	KAU	3	Private clinic	Outpatient	Pediatrics
xi1	28	F	KSU	4	General hospital (government)	Outpatient	Orthopedics/Sports
xi2	26	F	QU	<1	General hospital (government)	Inpatient	Cardiopulmonary

F, female; M, male; PT, physical therapy. Universities: QU, Qassim University; TU, Taif University; JU, Jazan University; UOH, University of Hail; IAU, Imam Abdulrahman Al Faisal University; KAU, King Abdulaziz University; KSU, King Saud University. Setting type “Mixed” = combined inpatient and outpatient. Participant IDs correspond to the codes used to attribute quotations in the main text and in Table 4.
