# Supplementary material for: Physical Therapists’ Self-Reported Perceptions of Bedside Manners in Saudi Arabia: A Mixed-Methods Study
Source: Healthcare (Basel). 2026 Jul 23;14(15):2250. doi: 10.3390/healthcare14152250 (PMC13465485; doi:10.3390/healthcare14152250)
Supplement: Supplementary file 1 [file healthcare-14-02250-s001.zip › Supplementary Table S2 - Q8 Q9 cross-tabs by work setting.pdf]

Table S2. Cross-tabulation of Section B Questions 8 and 9 by work setting (n = 147).

Question 8. "If the patient/client understands and knows his/her exercises, there is no need for me to be present with them during the session."

Work setting	Agree, n (%)	Neutral, n (%)	Disagree, n (%)	Total, n
Government hospital	7 (10.8%)	15 (23.1%)	43 (66.2%)	65
Private hospital	9 (30.0%)	5 (16.7%)	16 (53.3%)	30
Rehabilitation hospital	8 (47.1%)	2 (11.8%)	7 (41.2%)	17
Community care	0 (0.0%)	0 (0.0%)	1 (100.0%)	1
Private clinic	7 (20.6%)	3 (8.8%)	24 (70.6%)	34
All settings	31 (21.1%)	25 (17.0%)	91 (61.9%)	147

Question 9. "If the patient/client condition is minor, it is acceptable to offer him/her with less attention."

Work setting	Agree, n (%)	Neutral, n (%)	Disagree, n (%)	Total, n
Government hospital	4 (6.2%)	10 (15.4%)	51 (78.5%)	65
Private hospital	5 (16.7%)	5 (16.7%)	20 (66.7%)	30
Rehabilitation hospital	8 (47.1%)	1 (5.9%)	8 (47.1%)	17
Community care	0 (0.0%)	0 (0.0%)	1 (100.0%)	1
Private clinic	5 (14.7%)	3 (8.8%)	26 (76.5%)	34
All settings	22 (15.0%)	19 (12.9%)	106 (72.1%)	147

Five-point Likert responses were collapsed to three categories: Agree = strongly agree/agree; Neutral; Disagree = disagree/strongly disagree. Percentages are row percentages within each work setting. The single Community care respondent is shown for completeness but was not interpreted given the subgroup size.
